# Juglone in Oxidative Stress and Cell Signaling

**DOI:** 10.3390/antiox8040091

**Published:** 2019-04-05

**Authors:** Taseer Ahmad, Yuichiro J. Suzuki

**Affiliations:** 1 College of Pharmacy, University of Sargodha, Sargodha, Punjab 40100, Pakistan; drtasir2011@gmail.com; 2 Department of Pharmacology and Physiology, Georgetown University Medical Center, Washington, DC 20007, USA

**Keywords:** antioxidants, juglone, naphthoquinone, Pin1, redox, signal transduction, walnuts

## Abstract

Juglone (5-hydroxyl-1,4-naphthoquinone) is a phenolic compound found in walnuts. Because of the antioxidant capacities of phenolic compounds, juglone may serve to combat oxidative stress, thereby protecting against the development of various diseases and aging processes. However, being a quinone molecule, juglone could also act as a redox cycling agent and produce reactive oxygen species. Such prooxidant properties of juglone may confer health effects, such as by killing cancer cells. Further, recent studies revealed that juglone influences cell signaling. Notably, juglone is an inhibitor of Pin1 (peptidyl-prolyl *cis/trans* isomerase) that could regulate phosphorylation of Tau, implicating potential effects of juglone in Alzheimer’s disease. Juglone also activates mitogen-activated protein kinases that could promote cell survival, thereby protecting against conditions such as cardiac injury. This review describes recent advances in the understanding of the effects and roles of juglone in oxidative stress and cell signaling.

## 1. Introduction

Archaeological data suggest that the gathering and intake of walnuts by humans occurred as early as 7300 years ago in the Mediterranean [1]. The use of walnuts in traditional medicine suggests the presence of multiple, effective, and useful compounds which may provide health benefits such as antihypertensive, endothelial protective, anti-diabetic, and hepato-protective activities [2,3,4,5]. Walnuts contain various phytochemical constituents that may promote human health. The walnut is a dietary plant with one of the highest levels of antioxidants [6], and it has the highest level of phenolic antioxidants among nut species [7,8,9]. Walnut extracts have been found to contain flavonoids, terpenoids, gallic acid, caffeic acid, myricetin, and quercetin as well as naphthoquinones like juglone [10,11,12,13]. 

**Figure 1:**
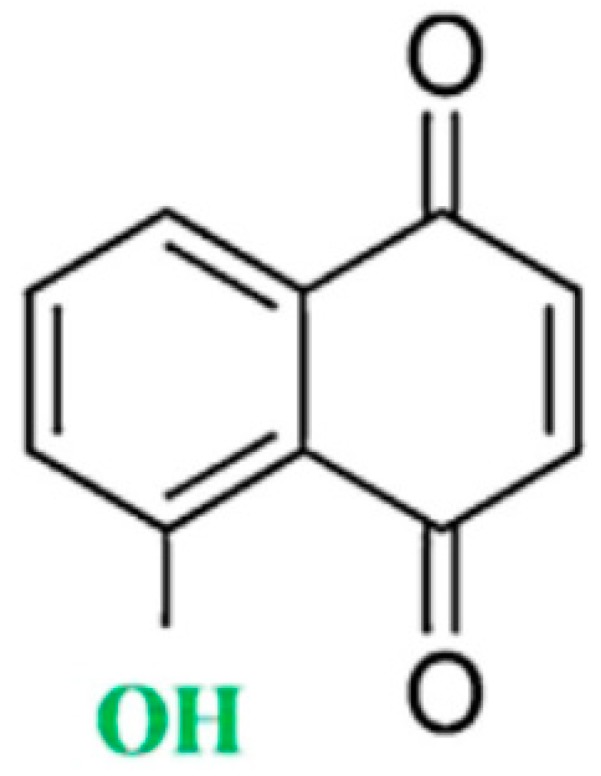
Chemical structure of juglone.

Juglone (5-hydroxy-1,4-naphtoquinone; see Figure 1 for the chemical structure) is found in the fresh ripe fruit husk, roots, leaves, and bark of walnut trees [14,15]. Juglone is produced by the numerous species of walnut tree including the *Juglans nigra* (black walnut), *Juglans regia* (English or Persian walnut), *Juglans sieboldiana* (Japanese walnut), and *Juglans cinerea* (butternut or white walnut) [16]. Juglone is also found in *Carya ovata* (hickory tree), Proteaceae [17], Caesalpiniaceae [18,19], and Fabaceae [20]. Most studies refer to the use of *Juglans nigra* for isolation of juglone and allelopathic studies because this particular species produces the largest amount of juglone [21]. 

It was not until the 1850s that juglone (then termed “nucin” from the Latin *nux*, meaning a nut) was first isolated from the walnut tree [22], and in 1881 the first scientific report on the allelopathic effect of juglone was published [23]. Further, juglone has been found to have various pharmacological actions, including a depressant effect [24], an impact on skin diseases [16], and antimicrobial [25], anti-cancer [26,27,28], anti-fungal [25], and antioxidant [29] activities, as well as apoptotic capacities [30] and anti-angiogenesis properties [31]. Juglone and its derivatives have been shown to possess the ability to inhibit food degradation by creating resistance to oxygen and its reactive species [29]. 

In this review, the redox properties of juglone in relation to its health benefits will be discussed. We also summarize the published results on the effects of juglone on cell signaling.

## 2. Antioxidant Properties of Juglone

**Figure 2:**
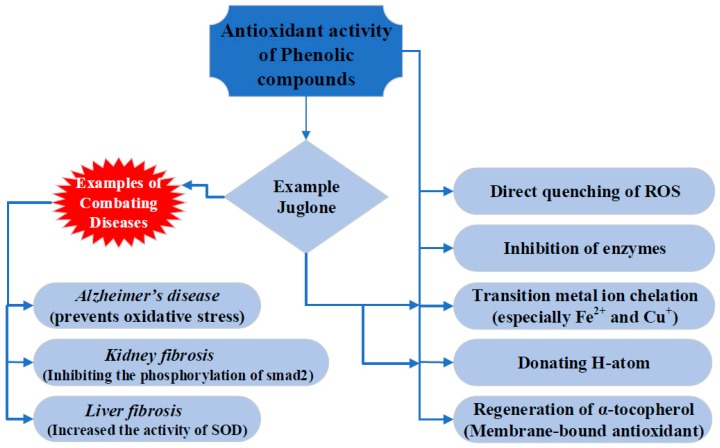
Schematic diagram describing the antioxidant properties of phenolic compounds including juglone.

Through the production of reactive oxygen species (ROS), biomolecules undergo oxidative stress. Antioxidants reduce ROS, and the balance between ROS and antioxidants defines oxidative stress. Phenolic compounds can inhibit these reactions by directly quenching ROS, inhibiting ROS producing enzymes, chelating transition metal ions, hydrogen atom transfer, and regeneration of vitamin E as depicted in Figure 2 [32,33,34]. The intramolecular hydrogen bonds play important roles in the stability of free radicals [33,35,36,37]. Juglone contains an intramolecular hydrogen bond between hydroxyl and keto groups and is active in donating the hydrogen-atom [38]. Juglone may have either pro- or antioxidant characteristics depending on the concentrations [29]. Thus, some studies reported the generation of ROS by juglone, while others describe its antioxidant properties [39]. 

Some antioxidants are capable of chelating transition metal ions (especially Fe^2+^ and Cu^+^) leading to the formation of stable complexes, thereby preventing these metals from participating in free radical generation [33,40,41,42]. Ferrous iron promotes lipid oxidation through Fenton reaction [43]. To avoid metal-catalyzed oxidation, the use of natural metal chelators instead of the synthetic counterparts should be encouraged [32]. It has been revealed experimentally that deprotonated juglone has the capacity to chelate Fe^2+^ [44]. 

Accumulating evidence suggest that antioxidant properties of juglone are useful in combating oxidative stress-linked diseases. Juglone has been shown to prevent oxidative and heat stress-induced dephosphorylation of Tau (an important step in the pathogenesis of Alzheimer’s disease) in in human cortical neurons [45]. A recent study in a transgenic mouse model of Alzheimer’s disease demonstrated that the walnut supplementation can reduce oxidative damage [46]. Oxidative stress is an important mechanism for kidney fibrogenesis [47,48], and Reese et al. reported that juglone reduces oxidative stress by inhibiting the phosphorylation of Smad2 in the kidney [49]. Zhou et al. [50] demonstrated that juglone increased the activity of superoxide dismutase and decreased oxidative stress in the liver. The authors also observed that juglone reduced the serum levels of alanine aminotransferase, aspartate amino-transferase, hyaluronic acid, laminin, type III procollagen, and type IV collagen and the expression levels of α-smooth muscle actin and collagen III in the liver [50].

## 3. Juglone as an Oxidant: Cytotoxic Potential 

The generation of ROS and modulation of redox signaling are properties of quinones that render them interesting leads for the development of novel compounds of potential use in various therapeutic sites [51].

Many quinoid compounds such as naphthoquinones have pharmacological and toxicological importance [52,53] because of their electrophilicity and redox properties. Naphthoquinones have been shown to possess cytotoxic [54,55], antitumor [56] and antimicrobial activities [25,57,58]. Naphthoquinones are among the most active natural products obtained from plants and microorganisms, and they exert their biological activities through pleiotropic mechanisms that include reactivity against cell nucleophiles, generation of ROS, and inhibition of proteins [59]. 

Juglone is a natural toxin produced by walnut trees [60]. Numerous proposals have been made to explain the mode of action underlying the cytotoxicity of juglone [61]. Although this molecule has multiple effects, including apoptosis [62], it is well known that these effects are cell-type-specific [60] and the exact mechanism remains unclear. According to Aithal et al. [63], the cytotoxic and genotoxic effects of juglone involve the induction of oxidative stress, cell membrane damage, and apoptosis and necrotic cell death. Supporting the theory that juglone causes oxidative stress, a small amount of juglone rapidly oxidizes a large amount of reduced nicotinamide adenine dinucleotide phosphate (NADPH) when added to rat liver microsomal preparations rich in one-electron reductases. This results in a large increase in oxygen consumption and ROS production. These events are prevented by pre-treatment with inhibitors of NADPH reductases [64]. Moreover, the cytotoxicity of juglone requires bioreduction to yield the semiquinone, which in turn reduces oxygen to superoxide [65].

Naphthoquinones, especially juglone are also considered as myotoxic quinones, which have been found to undergo rapid single-electron reduction [66]. This effect is also validated by the study in which the mammalian selenoprotein thioredoxin reductase 1 (TrxR1), a key enzyme in redox regulation, antioxidant defense, and cellular growth, catalyzes efficient reduction of juglone in a reaction. One-electron juglone reduction by TrxR1 produces superoxide and further contributes to the pro-oxidant cytotoxicity of juglone [67]. 

Some studies also reported that the cytotoxicity of juglone is due to two different mechanisms, namely, redox cycling and the reaction with glutathione (GSH) [14]. Redox cycling represents a cyclic process of reduction of a compound, followed by oxidation of the reaction product and the simultaneous generation of ROS [68]. Juglone enhances lipid peroxidation predominantly through redox cycling [69]. The second mechanism of the toxicity of juglone is the formation of adducts, which also causes the glutathione depletion. Juglone can also form adducts with nucleophiles via Michael-type addition to the quinone. The thiol group on reduced glutathione is a very good nucleophile, and it is easily arylated by juglone. Arylation of reduced glutathione by juglone increases cellular toxicity by decreasing the availability of reduced glutathione, an endogenous antioxidant [51,70,71].

Different studies confirmed the cytotoxic effects of juglone against the various types of human cell lines. Cytotoxic effects of juglone have been studied on human leukemia cell (HL-60 and HL-60R). The multidrug resistance developed by the doxorubicin-resistant HL-60 cell line did not prevent the cytotoxic effect of juglone [61]. Juglone exhibited cytotoxicity to human hepatoma cell line, HepG2, and the BALB/c mouse fibroblast cell line, 3T3 [72]. Juglone also differentially reduced the viability of human cells in culture through the induction of DNA damage, the inhibition of transcription, the reduction of p53 protein levels, and the induction of cell death [60]. Juglone exerted cytotoxic, anti-proliferative, and anti-invasive effects on C6 rat glioma cells *in vitro* [73].

Furthermore, the 5-hydroxy semiquinone free radical, superoxide, and hydroxyl radical (product of the Fenton reaction) have all been directly observed in human keratinocytes by electron paramagnetic resonance. Additionally, pretreatment with buthionine sulfoximine, an inhibitor of glutathione synthesis, decreases antioxidant availability and sensitizes cells to juglone toxicity [14]. The reverse of this is also true: pre-treatment of human leukemia cells (HL-60) with *N*-acetylcysteine, an antioxidant, prevents juglone-induced death [74]. Collectively, these results strongly support the importance of redox cycling and ROS in the cellular toxicity of juglone. However, it should be noted that the actions of antioxidants can be complex. Ascorbic acid (vitamin C) has recently been shown to potentiate the cytotoxicity of juglone by increasing the efficiency of redox cycling. Nevertheless, several decades of literature support the prevention of quinone toxicity by pretreatment with antioxidants. [75,76,77,78].

## 4. Anticancer Effects of Juglone

Quinones are plant-derived secondary metabolites that produce some anti-proliferation and anti-metastasis effects in various cancer types [79]. Several anti-cancer drugs contain the quinone nucleus and have proven useful in cancer chemotherapy [80]. Quinones are among the most frequently used drugs to treat human cancer. They undergo reversible enzymatic reduction and oxidation, and form semiquinones and oxygen radicals, thereby promoting oxidative stress and damage to tumor cells [81]. The antitumor activity of quinones is frequently linked to DNA damage caused by alkylating species or oxygen radicals [53]. Some important drugs used for the treatment of cancer belong to the quinone class of organic compounds, like daunorubicin and doxorubicin [82]. Natural quinones like 1, 4-naphthoquinones [83] seem to be promising for targeting cancer cells [84].

**Figure 3:**
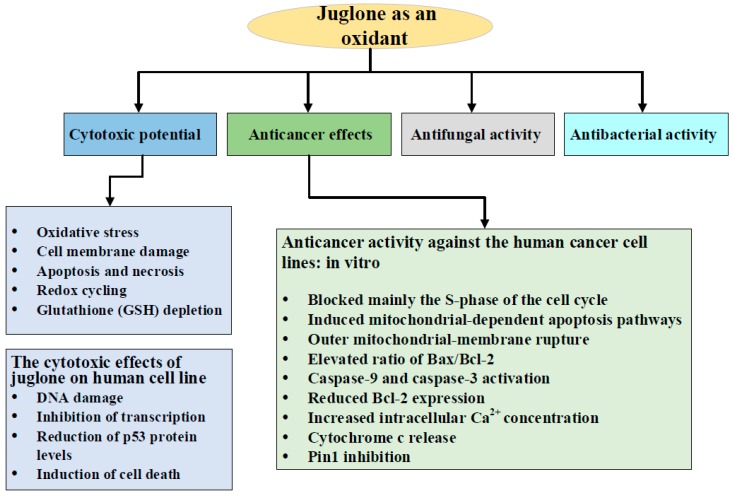
The schematic diagram showing the oxidant potential of juglone and its therapeutic applications.

The plant source of juglone, *juglans regia*, has been found to possess an anticancer capacity [85]. Thus, the isolated active chemical constituent, juglone, has been investigated in different human cancer cell lines (Figure 3). Since ROS play diverse roles in cancer, modulating the redox status of cancerous cells seems to be a promising therapeutic approach. The published data revealed that the cancer-related inhibitory effects of juglone are associated with enhanced ROS production and lipid peroxidation [78]. 

### 4.1. Anticancer Activity against the Human Cancer Cell Lines: in Vitro

Juglone has been studied for its growth inhibitory effects on cultured malignant cells such as HCT-15 cells derived from human colon carcinoma and was found to block mainly the S phase of the cell cycle [86]. Juglone induces apoptosis in HL-60 human leukemia cells [87], SGC-7901 human gastric cancer cells [88] and SKOV3 ovarian cancer cells [89] through mitochondrial-dependent apoptosis pathways and the elevated ratio of Bax/Bcl-2. The anti-cancer activity on LNCaP human prostate cancer cells indicated that juglone may be a potential candidate drug for androgen-sensitive prostate cancer [90]. The cytotoxic effect of juglone on human breast cancer cell line MCF-7 is characterized by elevated ROS levels, reduced Bcl-2 expression, increased Bax expression, decreased mitochondrial membrane potential, increased intracellular Ca^2+^ concentration, outer mitochondrial-membrane rupture, cytochrome c release, and caspase-3 activation [91]. According to a recent study, juglone significantly inhibits the proliferation and induces the apoptosis of human bladder carcinoma cell lines (TCC-SUB and RT-4) [92].

### 4.2. Glioma Cells

Glioma is a type of tumor that starts in the glial cells of the brain or the spine [93]. Gliomas comprise about 30 percent of all brain tumors and central nervous system tumors, and 80 percent of all malignant brain tumors [94]. The available data indicates that peptidyl-prolyl *cis/trans* isomerase Pin1 is overexpressed in human glioblastoma multiforme specimens. Therefore, Pin1 inhibitors should be investigated as a new chemotherapeutic drug that may enhance the clinical management of human gliomas. Recently, juglone, a Pin1 inhibitor, was shown to exhibit potent anticancer activity in various tumor cells including U251 glioma cells, and it was observed to disrupt angiogenesis [31]. Juglone also exhibits the anticancer effects in glioma C6 cells by generating ROS through interaction with respiratory complex I [95]. 

### 4.3. In Vivo Anticancer Effects of Juglone

The effect of juglone on intestinal carcinogenesis in rats was examined through dietary exposure during the initiation phase. The data suggest that juglone could be a promising chemopreventive agent for human intestinal neoplasia [26]. Tumor progression in mice is inhibited by juglone, which triggers oxidative stress that leads to apoptosis and cell cycle detention, the suppression of hypoxia-inducible factor-1 alpha, and separation of glycolytic metabolism [77]. Therefore, additional studies are warranted to examine the clinical potential of juglone in human cancers.

## 5. Antimicrobial Activity of Juglone

### 5.1. Antifungal Activity 

The juice of freshly macerated unripe hulls of the black walnut (*Juglans nigra*) has been used for many years in folk medicine as a treatment for localized, topical fungal infections [25,96]. The compound that may be responsible for the wide range of biological activities of *Juglans nigra* is juglone [25]. Wianowska et al. also showed the antifungal activity of juglone and walnut green husk extracts [97]. Juglone may be as effective as commercially available antifungal agents including zinc undecylenate and selenium sulfide [25]. 

### 5.2. Antibacterial Activity 

Juglone has been shown to possess antibacterial activities [57,58], and reportedly inhibits Gram-positive bacteria, *Staphylococcus aureus* [98]. Juglone exhibits a selective antimicrobial activity against different sources of *Staphylococcus aureus* strains. Juglone has been considered to be a natural source for resistance-modifying activity in the same bacteria and as phytochemical constituents with antibiotic resistance-modifying activity [99]. It is also likely that the mechanisms of antifungal and antibacterial actions of juglone involve redox cycling [14]. A bioinformatics analysis has shown that bacterial proteins that participate in DNA, RNA and protein synthesis as well as in the tricarboxylic acid cycle are inhibited by juglone [98]. It has also been reported that the antifungal and antibacterial activities of juglone nanoparticles are higher than those of free juglone, indicating that the nanoparticle formulation may be a promising way to enhance the beneficial effects of juglone [100,101]. 

## 6. Role of Juglone as an Inhibitor of Peptidyl-Prolyl *cis/trans* Isomerase, Pin 1 

Juglone is often used as an inhibitor of peptidyl-prolyl *cis/trans* isomerases that catalyzes the *cis/trans* isomerization of peptide bonds preceding prolyl residues [102]. Peptidyl-prolyl *cis/trans* isomerases can regulate protein phosphorylation and cell signaling [103]. Since the identification of the importance of human peptidyl-prolyl *cis/trans* isomerase Pin1 in Alzheimer’s disease [104] through the modulation of Tau protein [45], Pin1 and juglone have gained considerable attention. As a result, and largely through studies using juglone, Pin1 has also been implicated in a wide variety of clinical conditions including immune response [105], allergy [106], cancer [107,108], hyperparathyroidism [109], rheumatoid arthritis [110], vascular pathology [111,112,113,114,115], diabetes [116], Parkinson’s disease [117], and cardiac fibrosis [118,119,120]. There are numerous review articles already available on Pin1, and thus interested readers should refer to these articles that are found in PubMed and other sources.

## 7. Effects of Juglone on Cell Signaling Pathways

In addition, juglone has been reported to influence a variety of other cell signaling pathways. It was found to activate MAP kinases including ERK, JNK and p38 in skin cells [121], glioblastoma cells [122], cervical cancer cells [123], melanoma cells [30], hepatocellular carcinoma cells [124], and smooth muscle cells [125]. In particular, cell signaling modulations of MAP kinases by juglone have been attributed to the mechanism of the induction of apoptosis. Some studies have shown that the MAP kinase activation is dependent on the production of ROS [30,122,123,124], suggesting that the redox cycling of naphthoquinones may be responsible for the mechanism.

In contrast to the activation of MAP kinase pathways, juglone has been shown to suppress the Akt pathway. Fang et al. [28] reported that, in prostate cancer cells, juglone inhibits the Akt/GSK-3b/Snail pathway and the subsequent epithelial-mesenchymal transition process. Similarly, juglone potentiates the reduction of Akt phosphorylation induced by high glucose, leading to suppressed eNOS-dependent nitric oxide pathway in rat aortas [28,126]. Juglone was also found to reduce the Akt phosphorylation level in breast cancer cells that are resistant to the trastuzumab anti-cancer monoclonal antibody [127].

Another interesting effect of juglone is its ability to activate the Ca^2+^ channel activity of transient receptor potential ankyrin subtype 1 channel, TRPA1 [128]. TRPA1 is an irritant sensor and plays a key role in nociception, irritant sensing and mechanical sensation [129]. These authors showed that juglone and plumbagin (both of which possess a hydroxyl group at the 5 position), but not lawsone (that has a hydroxyl group at the 2 position), exhibited the agonist effects. Thus, the hydroxyl group at the 5 position likely plays a mechanistic role in this activity. Further, since neither extracellular nor intracellular application of catalase (a hydrogen peroxide scavenger) prevented the activation [128], it is not likely that redox cycling-mediated ROS production is involved in this mechanism. 

## 8. Conclusions

Juglone, a phenolic compound found in walnuts, has been shown to exert both oxidant and antioxidant activities, to act as an inhibitor of Pin1, and to modulate cell signaling. These diverse actions may confer the possible health benefits of walnuts. Further, juglone may be useful as a therapeutic agent to combat various diseases and to promote health. Further studies are needed to examine the clinical potential of juglone. One limitation of the juglone research field is that published studies have not performed comprehensive structure-activity relationships. Therefore, it is unclear whether the effects of juglone are specific or are shared by other naphthoquinone molecules. Nevertheless, juglone is an exciting molecule both chemically and biologically, and we hope that this review article will help future research.
